# Kinetics and Thermodynamics Study on Removal of Cr(VI) from Aqueous Solutions Using Acid-Modified Banana Peel (ABP) Adsorbents

**DOI:** 10.3390/molecules29050990

**Published:** 2024-02-24

**Authors:** Zhouyang Huang, Robyn Campbell, Chirangano Mangwandi

**Affiliations:** School of Chemistry and Chemical Engineering, Queen’s University Belfast, David Kier Building, Stranmillis Road, Belfast BT95AG, Northern Ireland, UK

**Keywords:** banana peel, chromium, desorption, wastewater, reduction, sulfuric acid

## Abstract

Banana peel waste is abundant and can be utilized as a low-cost adsorbent for removing toxic Cr (VI) from wastewater. The acid modification of banana peels significantly enhances their adsorption capacity for Cr (VI). An adsorbent was prepared by treating banana peel powder with 50% H_2_SO_4_ at 50 °C for 24 h. The acid treatment increased the surface area of the adsorbent from 0.0363 to 0.0507 m^2^/g. The optimum adsorbent dose was found to be 1 g/L for the complete removal of Cr (VI) from 100 ppm solutions. The adsorption capacity was 161 mg/g based on the Langmuir isotherm model. The adsorption kinetics followed a pseudo-second order model. Increasing the temperature from 20 to 50 °C increased the initial adsorption rate but had a minor effect on the equilibrium adsorption capacity. Thermodynamics studies showed that the process was spontaneous and endothermic. The activation energy was estimated as 24.5 kJ/mol, indicating physisorption. FTIR analyses before and after adsorption showed the involvement of hydroxyl, carbonyl and carboxyl groups in binding the Cr (VI). The Cr (VI) was reduced to Cr (III), which then bound to functional groups on the adsorbent. Desorption under acidic conditions could recover 36% of the adsorbed Cr as Cr (III). No desorption occurred at a neutral pH, indicating irreversible adsorption. Overall, acid-modified banana peel is an efficient, low-cost and eco-friendly adsorbent for removing toxic Cr (VI) from wastewater.

## 1. Introduction

According to UNESCO, in 2023, globally, approximately 2 billion people (26% of the population) do not have access to safe drinking water [1]. Furthermore, a study released in 2017 called *Global Burden of Disease* discovered that polluted water is responsible for 1.2 million deaths each year [2]. Due to the rise in the levels of human and industrial activities, heavy metals in drinking water are still not at the recommended limits. Such heavy metals found commonly in water include arsenic, chromium, lead, manganese and copper, and when ingested in small quantities, these heavy metals can be toxic. 

Chromium is naturally found in rocks, plants, soil and volcanic dust. However, due to its heavy use in industrial activities such as metal finishing, leather tanning, textile production and dyeing, chromium in various forms is found in the atmosphere and in water bodies. This leads to a serious threat to human life and environmental toxicity [3]. Chromium exists in the following two main forms: hexavalent chromium (Cr (VI)) and trivalent chromium (Cr (III)). Trivalent chromium is found to cause damage to the brain, liver and kidneys in large amounts when ingested orally. However, hexavalent chromium is more toxic to soil, plants, animals and humans than trivalent chromium. Cr (VI) exposure causes reduced soil fertility and affects plant enzyme and photosynthesis, and when directly exposed to humans, it results in poisoning, cancer and cellular damage [3]. Hence, proactive measures are imperative for attaining and upholding the Environmental Protection Agency’s (EPA’s) stipulated maximum limit of 100 µg/L for chromium in drinking water.

Several methods have been conventionally used to remove heavy metals from wastewater such as membrane-based filtration, precipitation, coagulation and electric-based separation methods such as ion exchange. However, many of these methods incur high costs and produce large volumes of sludge [4]. Therefore, adsorption is becoming an increasingly popular heavy metal removal method due to its higher sustainability and the lower costs associated with the process. Biosorption is a type of adsorption process in which contaminants bind onto the surface of a biomass such as food waste [5,6,7], wood, wood residues [8], agricultural residues [9] and household wastes. According to Statista, bananas are one of the most widely produced and consumed fruits in the world, with production levels as high as ~125 million metric tons for the year 2021 [10]. Bananas are known as a tropical fruit which is grown in the tropical regions of Africa, Latin America, the Caribbean and the Pacific. In 2020, the top three countries exporting bananas were Ecuador (33%), the Philippines (18%) and Costa Rica (12.5%) [11] due to their ideal climates for banana growth, which include sunshine between 9 and 15 months of the year and temperatures near 27 °C. However, due to the high production and consumption of bananas, large volumes of banana waste are produced annually. Banana peels, which make up ~35% of bananas by weight, produce 3.5 million tons of waste per year. This is detrimental to the environmental as this waste is usually sent to landfills and/or burnt, which results in the release of greenhouse gases which include methane and carbon dioxide, contributing to global warming [6]. However, banana peels are rich in nutrients and minerals, containing 6–9% protein and 20–30% fiber, and green banana peels contain ~15% starch while ripe banana peels contain up to 30% free sugars [12]. This makes banana peels an ideal feedstock for several applications such as agricultural feedstock for cattle, goats and other animals; culinary use in recipes in parts of the world such as Southeast Asia; water purification; and use as a fertilizer.

Banana peel use for wastewater treatment is becoming increasingly popular, and several researchers have studied this subject, such as Varney Edwin Johnson et al., who modified banana peels via hydrothermal synthesis for Pb(ii) and As(V) removal from wastewater [13], Adhi Setiawan et al., who used banana peels to develop Fe-impregnated activated carbon for the removal of methylene blue from wastewater [14], and K.M. Lavanya et al., who used raw and alkali metal-free banana peels to remove Hg^2+^ ions from wastewater [15]. Pristine banana peel has also been applied as an adsorbent for the removal of chromium, achieving capacities ranging from 90 to 130 mg/g [16,17]. Chen et al. carried out a preliminary investigation on the effects of activation methods on the chromium removal capacity of the resultant adsorbent, and their study proved that acid treatment increased from 90 to 163 mg/g (approximately 77%) [16]. The objective of this research is to extend earlier work—in [16]—which acid treatment was used to enhance the adsorption capacity of banana peel adsorbent. The current work includes a detailed study on the kinetics of the process and investigated the effect of temperature on the kinetics of the removal process, with a detailed characterization of the adsorbent before and after removal of the chromium to elucidate on the chromium removal mechanism. In this work, the adsorbent from banana peel was synthesized and surface morphological studies were conducted using FTIR and SEM to relate the adsorbent’s characteristics to its adsorption ability.

## 2. Results and Discussion

### 2.1. Characterization

#### 2.1.1. BET Analysis

The surface area of both the untreated banana peel and the banana peel subjected to sulfuric acid modification was determined through BET analysis, with the results summarized in Table 1. Remarkably, the surface area of the banana peel significantly increased by nearly 1.5 times after modification with sulfuric acid. The increase in the surface area may have partially contributed to the observed increase in the removal capacity of the acid-modified banana peel adsorbent. This notable enhancement could be attributed to the composition of a natural banana peel, which is rich in cellulose, lignin and pectin, all of which feature abundant hydroxyl groups. These macromolecular structures foster the formation of numerous hydrogen bonds, both intermolecular and intramolecular.

The mechanism behind this transformation involves the impact of sulfuric acid at elevated temperatures. Firstly, the acid disrupts the hydrogen bonds between molecules in the banana peel, causing the separation of lignin and cellulose into distinct entities. Simultaneously, the acidic conditions facilitate the condensation of tannins, leading to solidification and the formation of larger cage-like molecules within the banana peel [18]. This dual action underlined the significant increase in surface area observed in the acid-modified banana peel.

#### 2.1.2. Elemental Analysis

The elemental analysis results for the banana peel samples subjected to varying concentrations of sulfuric acid are presented in Table 2. Notably, no significant disparity was observed in the hydrogen and carbon contents between the two samples. Nevertheless, the nitrogen content in the acid-modified banana peel (ABP) was markedly lower than that in the unmodified banana peel (BP). The sulfuric acid treatment resulted in a substantial increase in the sulfur content in the ABP sample. It is noteworthy that the carbon and nitrogen contents of both samples closely aligned with previously reported values in the literature, specifically, 35.65% for carbon and 1.94% for nitrogen [19].

#### 2.1.3. Functional Group Analysis Using FTIR

**Figure 1:**
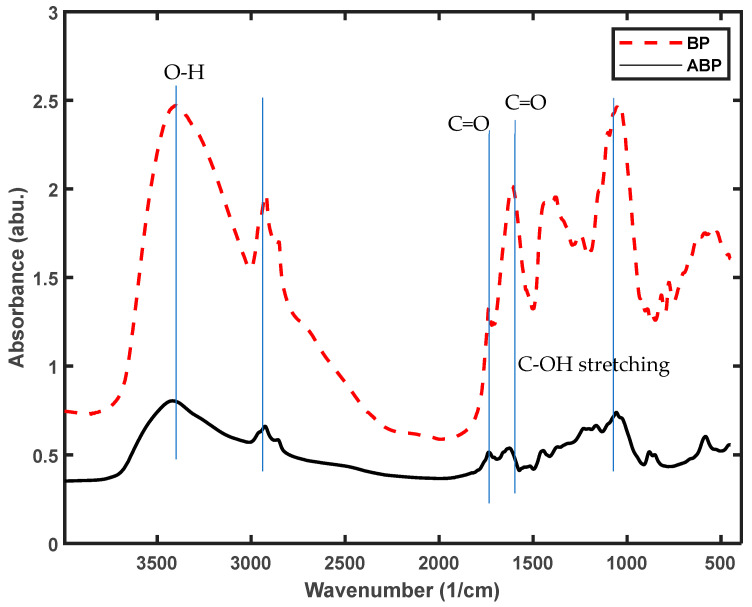
FTIR spectra of the BP and ABP samples before adsorption of the metal ions.

The impact of acid modification on the presence of functional groups in the sample was assessed through FTIR analysis, and the findings are presented in Figure 1. Examination of the results shown in Figure 1 reveals a significant peak variation within the 1000–2000 cm^−1^ range, indicating that the modification primarily influenced the methoxy groups (-OCH_3_) in lignin molecules and the hydroxyl groups in phenolic compounds (-OH). These two functional groups play a crucial role in the formation of hydrogen bonds, both intermolecular and intramolecular. Consequently, it could be tentatively concluded that the modification had achieved its intended purpose. Detailed changes in specific peaks are provided in Table 3.

#### 2.1.4. Textural Analysis

**Figure 2:**
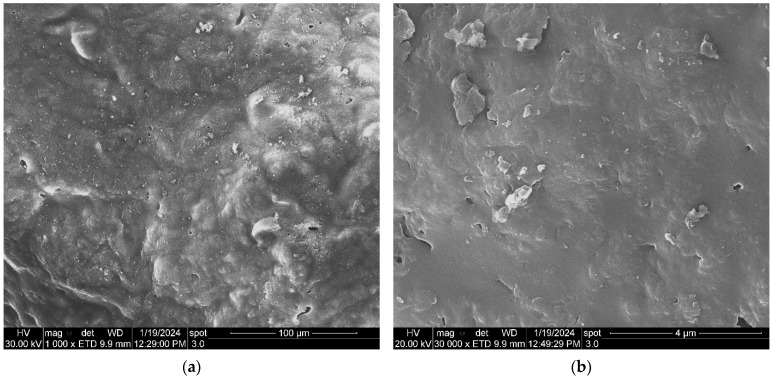
SEM images of the ABP sample before adsorption of the Cr(VI): 1000× magnification (**a**), and 30,000× magnification (**b**).

The SEM measurements allowed for an investigation of the morphology of the adsorbent synthesized from the banana peel (ABP) before adsorption. Figure 2a,b shows that at magnifications of 1000 and 30,000, respectively, the surfaces of the adsorbents appeared to be rough and sponge-like, with visible pores, which made them ideal for adsorption. It also shows that the surface of the adsorbent was rough.

**Figure 3:**
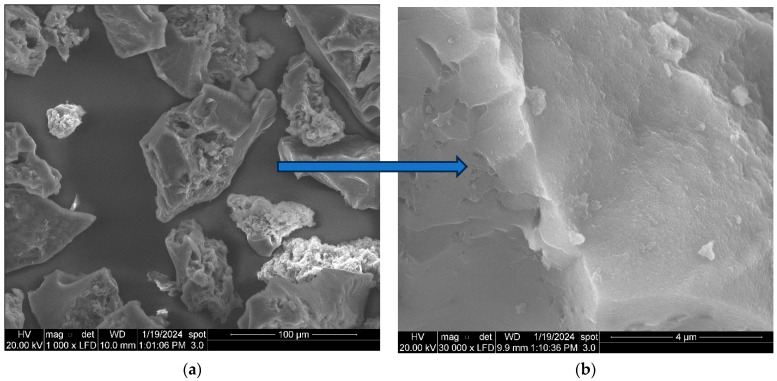
SEM images of the ABP sample after adsorption of the Cr(VI): 1000× magnification (**a**), and 30,000× magnification (**b**).

The textural properties of the adsorbent after adsorption of the Cr were also evaluated through SEM. The surface structure of the adsorbent did not significantly change after the adsorption process. Figure 3a,b shows SEM images of the ABP sample after Cr adsorption at magnifications of 1000 and 30,000, respectively.

**Figure 4:**
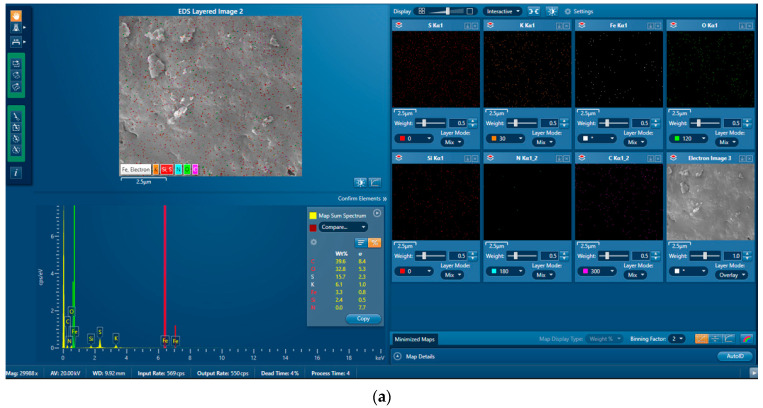

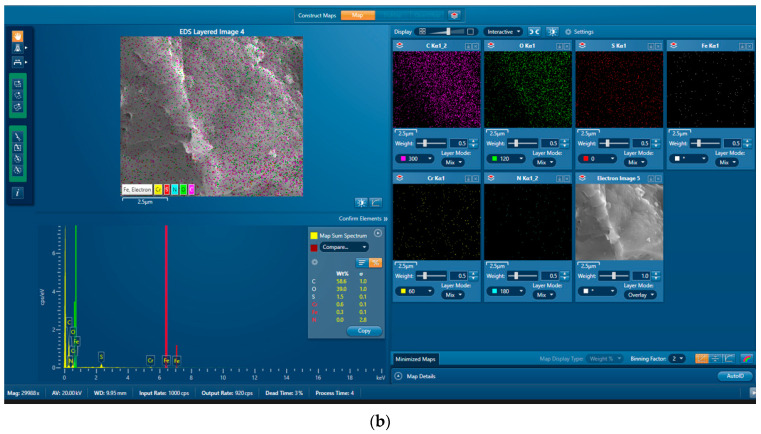
Distribution of the main elements detected during the SEM-EDX analysis of the ABP after adsorption of the hexavalent chromium. (**a**) before adsorption of Cr(VI); (**b**) after adsorption of Cr(VI).

The distribution of the elements on the surface of the adsorbent before and after adsorption is shown in Figure 4a,b. The XPS results of the sample before adsorption showed no presence of chromium, and approximately 0.6% Cr was observed on the surface of the sample after adsorption. This confirmed the successful loading of the chromium. However, it was noted that the Cr content was low. This could have been due to most of the Cr being loaded into the pore structure of the adsorbent. To further the successful loading of the chromium, the spent adsorbent collected after the adsorption process was digested in acid and the concentration of Cr present in the solution was analyzed using ICP. Higher levels of chromium were detected in the digested spent adsorbent. A comparison of the elemental composition of the sample before and after adsorption is presented in Table 4.

### 2.2. Determination of the Optimum Dose of ABP

**Figure 5:**
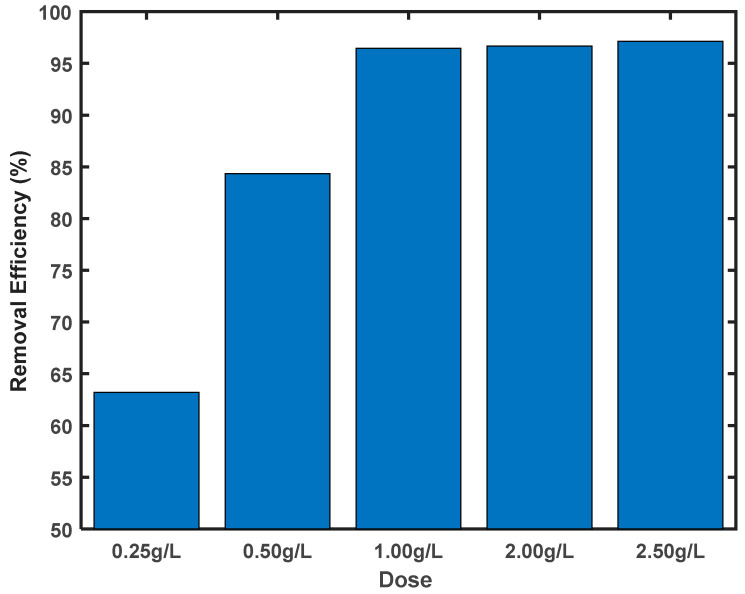
Effect of the ABP dosage on removal efficiency. The initial concentrations of the solutions were 100 ppm, and the pH levels of the solutions were adjusted to 2. The volumes of the solutions were 20 mL and the contact time was 72 h.

The removal efficiency was calculated from the residual concentration, and the results are presented in Figure 5.

The removal percentage of the Cr (VI) was close to 100% when 0.02 g or more of the ABP sample was used. Increasing the mass of the ABP beyond 0.02 g did not result in further increases in removal efficiency, implying that the optimum dosage was 1 g/L (i.e., 0.02 g in 0.02 L). The optimum dosage obtained here was comparable to results in the literature [20,21].

### 2.3. Evaluation of the Adsorption Capacity

**Figure 6:**
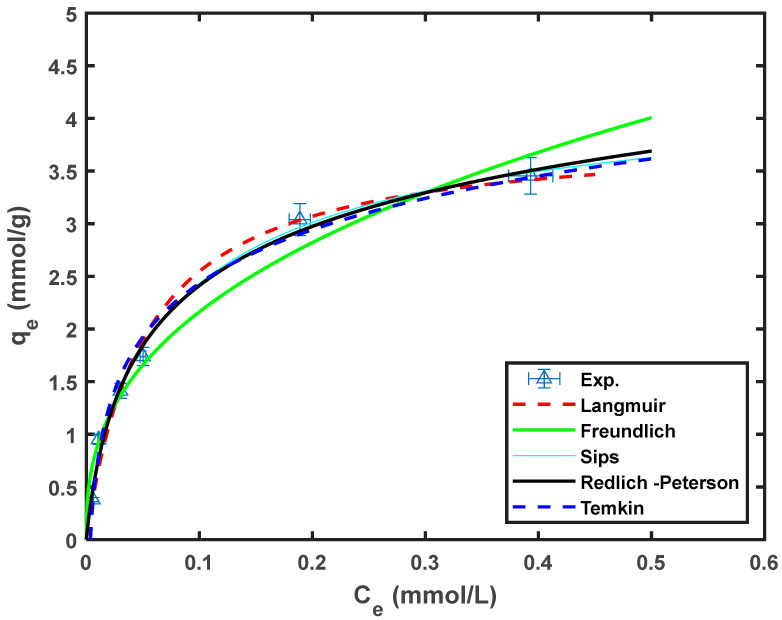
Fitting of the different isotherm models to the experimental data.

Non-linear regression fits of Equations (3)–(7) were performed using a custom-written MATLAB code. The parameters of the various isotherms are summarized in Table 5, and the fit of the model to the experimental data is shown in Figure 6.

Based on the preliminary experiments, the BP had a Langmuir adsorption capacity of approximately 90 mg/g, and the adsorption capacity increased to over 163 mg/g after modification [16]. Therefore, it could be concluded that the adsorption capacity of the banana peel could be enhanced by acid modification. A comparison of the adsorption capacities of Cr^6+^ adsorbed onto different bio-adsorbents reported by other researchers is shown in Table 6.

The results suggested that the modified banana peels had high adsorption capacities for Cr^6+^; therefore, they could be used as low-cost bio-adsorbents for the treatment of Cr^6+^-contaminated wastewater. From the FT-IR results, it was found that −OH, −C=O’ and −COO− were the main functional groups in all the BP and ABP samples. Therefore, the differences in the adsorption capacities could have been related to the quantities of these functional groups.

### 2.4. Adsorption Kinetics at Different Temperatures

**Figure 7:**
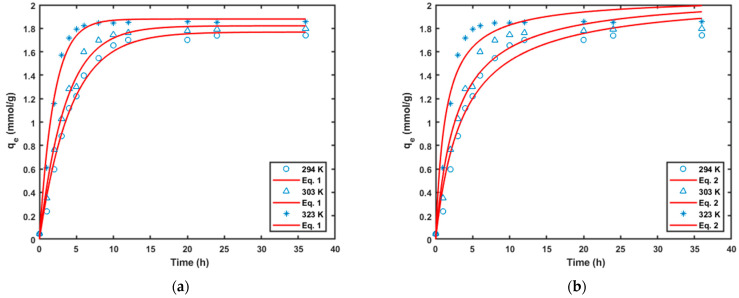
Fit of the different models for the Cr(VI) removal kinetics data at different temperatures: (**a**) pseudo-first-order model, and (**b**) pseudo-second-order model.

The adsorption kinetic study was conducted at three different temperatures (20, 30 and 50 °C), respectively, at the same initial pH, feed amount and Cr (VI) concentration. The results are shown in Figure 7, and it could be concluded that the rise in temperature accelerated the adsorption time to equilibrium. The results showed that a higher temperature was more conducive to Cr^6+^ reduction and adsorption in the adsorption process, as this was an endothermic reaction. The agreed with previously reported results [23,25,26]. The removal process was initially rapid, followed by a second-stage rate that gradually reduced to zero due to increased resistance to diffusion. The plots show that the initial removal rate of the Cr (VI) increased as the temperature increased. Different kinetic models were evaluated for their effectiveness in describing the removal of the Cr (VI) from the solution.

The removal of the Cr (VI) was examined using kinetic models, i.e., pseudo-first-order (PFO) and pseudo-second-order (PFO) kinetics. The pseudo-first-order kinetics model is expressed as follows:

(1) $$q_{t}=q_{max}\left(1-exp\left(-k_{1}t\right)\right),$$

where *q_t_* is the amount of solute adsorbed at the time *t* (min), *q_e_* is the amount of solute (mg g^−1^) adsorbed at saturation and *k*_1_ is the pseudo-first-order rate constant (h^−1^).

The pseudo-second-order kinetics model is generally expressed as follows:

(2) $$q_{t}=\frac{k_{2}q_{e}^{2}t}{1+k_{2}q_{e}t},$$

where *q_e_* is the amount adsorbed at equilibrium (mg/g) and *k*_2_ (g min^−1^ mg^−1^) is the pseudo-second-order rate constant.

The appropriateness of the models was evaluated using R^2^ and RSME. A custom written MATLAB code was written to obtain the non-linear regression fit, and the model’s parameters are reported in Table 7.

The curve-fitting results showed that both PFO and PSO were appropriate for describing the removal kinetics of the Cr (VI); however, the PFO had better goodness of fit values. The results also showed that increasing the temperature from 293 to 323 K did not have a significant effect on the removal capacity as the q_max_ values were not significantly different. However, the initial removal rate of the Cr(V), h, was significantly influenced by the temperature. The initial removal rate increased from 0.562 to 1.585 as the temperature of the adsorption was increased from 20 to 50 °C. The trends reported here are similar to what has been reported in the literature. For instance, in recent work on the removal of arsenic by adsorption onto magnetic bleached biochars (MBBC), increasing the temperature from 40 to 60 °C resulted in an increase in the initial rate of adsorption from 3.29 to 7.23 mmol g^−1^ min^−1^ [26]. A similar trend was also reported by Jaiyoela et al. in their work on the removal of Cr(VI) ions from an aqueous solution by CeO_2_@starch nanoparticles [23].

#### 2.4.1. Thermodynamics

To assess the potential adsorption mechanism of the Cr(VI) on the adsorbent ABP material, we utilized the adsorption data collected at the various temperatures. These data were employed to determine the thermodynamic parameters for the removal of the Cr(VI) by ABP, including the standard Gibbs free-energy change (Δ*G*°), enthalpy change (Δ*H*°) and entropy change (Δ*S*°). These parameters were calculated using the following equations:

(3) $$\Delta G{}^{\circ}=-RTlnK_{L}^{0}\mathrm{and}$$

(4) $$lnK_{L}^{0}=\frac{\Delta S{}^{\circ}}{R}-\frac{\Delta H{}^{\circ}}{RT}.$$

In these equations, R (8.314 J/mol K) represents the universal gas constant, T (K) denotes the absolute temperature and $K_{L}^{0}$ stands for the equilibrium constant at the temperature T, which is given by the following [27]:

$$K_{L}^{0}=\frac{\frac{q_{e}}{q_{m}}}{1-\left(\frac{q_{e}}{q_{m}}\right)\frac{C_{e}}{C_{o}}},$$

where $C_{e}$ (mol/L) signifies the equilibrium concentration of the Cr(VI) solution, $q_{e}$ (mmol/g) represents the amount of Cr(VI) removed by the ABP at equilibrium, $q_{m}$ (mmol/g) is the maximum adsorption capacity and $C_{o}$ (1 mol/L) is the reference standard concentration.

All the calculated thermodynamic parameters are provided in Table 8. As indicated in the table, both ΔH° and ΔS° exhibited positive values during the removal of the Cr (VI) by the ABP. The positive ΔH° values indicated an endothermic removal process, while the positive ΔS° values suggested an increase in disorder or randomness at the solid–solution interface [28]. Because the value of the enthalpy of the adsorption was within the range of 20 to 40 kJ/mol, it could be postulated that the removal of the Cr (VI) from the solution may have been mainly due to the physical adsorption of the di-chromate ions onto the solid surface by weak Van der Waals forces [29].

In the case of Cr (VI) removal by the ABP, all ΔG° values were found to be negative, and these values decreased as the adsorption temperature increased. This observation implied that the process was spontaneous, and its spontaneity was further enhanced at higher temperatures. This trend aligns with findings reported in the literature for various materials [24,28,30,31]. It has been shown in the literature that a ΔG value of below 18 kJ/mol (absolute values) indicates that the removal mechanism is dominated by physisorption [32].

#### 2.4.2. Determination of the Activation Energy

**Figure 8:**
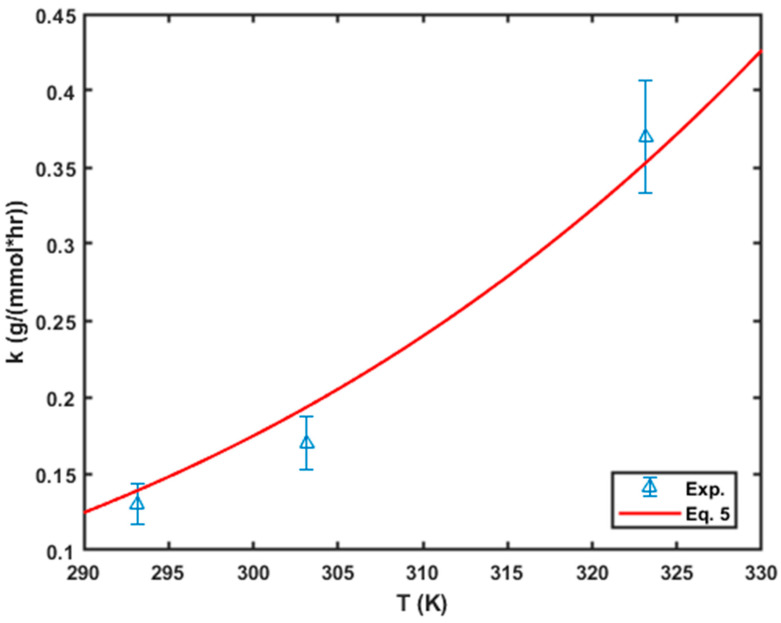
Variation in the rate of adsorption with the adsorption temperature.

The temperature dependence of the rate of chromium removal can be described by the Arrhenius equation, which is expressed as follows:

(5) $$k=Aexp\left(-\frac{E_{a}}{RT}\right),$$

where $E_{a}$ is the activation energy (J/mol), k is the rate constant of the adsorption, A is the frequency factor, R is the universal gas constant (8.134 J/mol) and T is the adsorption temperature (K). The rate constant from the PSO equation was plotted as function of the temperature, and the Arrhenius equation was fitted to the data using non-linear regression to obtain the activation energy for the Cr (VI) removal process (see Figure 8). The activation energy for the adsorption process was found to be 24.5 kJ/mol, which was almost half the value reported by Cantu et al. in their work on the removal of Cr (VI) from a solution by manganese oxide particles [33]. Based on the obtained value of the activation energy, it could be deduced that physisorption could be one of the possible removal mechanisms since the activation energy was less than 40 kJ/mol [33].

### 2.5. Adsorption Mechanism Discussion

**Figure 9:**
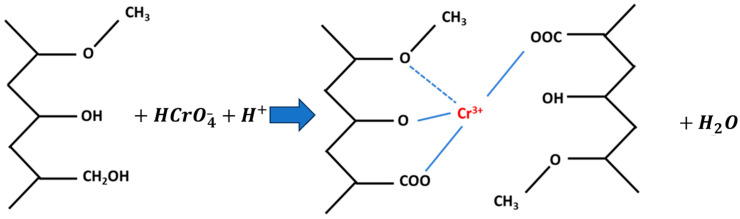
Reaction between Cr(VI) and lignin molecules in banana peel.

**Figure 10:**
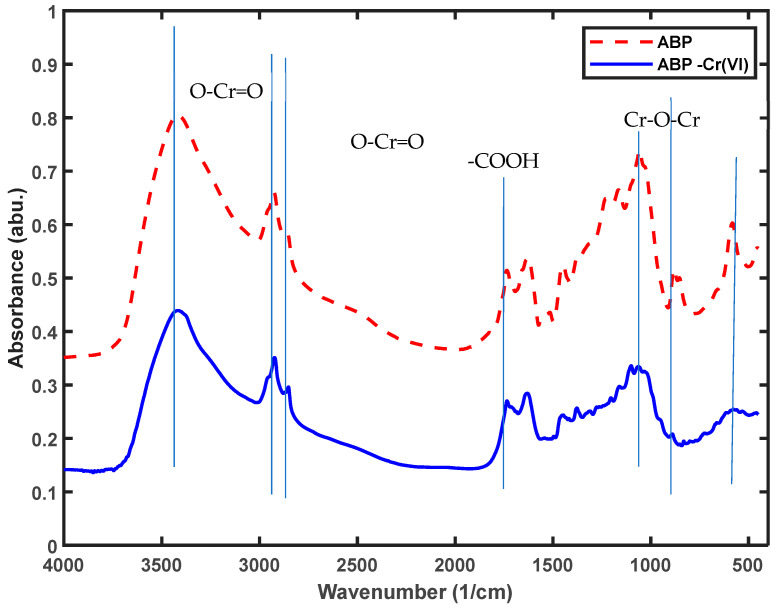
Comparison of the FTIR samples before and after adsorption of the Cr(VI).

Banana peel comprises insoluble components such as hemicellulose, lignin, cellulose and pectin [34,35], along with some reductants capable of reducing Cr (VI) to Cr (III) under acidic conditions [17,36]. These reductants include polysaccharides, glycoproteins, glucolipids and nucleic acids. The lignin within banana peel may play a role in Cr^3+^ removal, as depicted in the schematic reaction shown in Figure 9. The changes in the functional groups after adsorption are shown Figure 10.

Figure 10 indicates that the main functional group changes before and after adsorption are concentrated between the hydroxyl group, methoxy group and carbonyl group, with partial peaks shifting and disappearing and new peaks forming, indicating that during the adsorption process, the reduction in Cr (VI) causes the oxidation of some functional groups on the adsorbent and the increases in -C=O and –COOH (the peaks near 1730 cm^−1^). The peaks near 940 cm^−1^ and 1147 cm^−1^ represent the Cr-O-Cr bond, which provides further confirmation of the interaction of Cr with the adsorbent after adsorption [37,38,39].

This shift signifies alterations in functional groups due to Cr (VI) adsorption, involving the reduction in Cr (VI) causing the oxidation of certain functional groups on the adsorbent. The increased presence of -C=O and –COOH suggests a subsequent combination of reduced Cr (III) with carboxyl hydroxyl groups, leading to adsorption on the adsorbent’s surface.

In summary, chromium removal by the ABP adsorbent involves: (1) the direct adsorption of Cr(VI) in the form of hydrated anions at positively charged sites; (2) the reduction of Cr(VI) to Cr(III) by lignin and other reductive molecules under acidic conditions; and (3) the adsorption of Cr(III) on the adsorbent surface as a complex. The reduced Cr (III) is combined with the carboxyl hydroxyl group and adsorbed on the surface of the adsorbent.

### 2.6. Desorption Study

No chromium was detected in the distilled water; however, in the acidic conditions, only trivalent chromium was detected, with no hexavalent chromium. The desorption rate under acidic conditions was found to be 36%. The results showed that adsorption of the Cr (VI) was irreversible under neutral pH conditions. This was a good result as it implied that there was no chance of the Cr (VI) leaching back into the aqueous phase without pH adjustment once it was removed. On the other hand, the low desorption rate would have implications about the recyclability of the adsorbent material.

## 3. Materials and Methods

### 3.1. Materials

Potassium dichromate (K_2_Cr_2_O_7_), purchased from Sigma-Aldrich,(Gillingham, United Kingdom), was used to prepare the Cr(VI) stock solution. Hydrochloric acid (HCl) and sodium hydroxide (NaOH), both supplied by Sigma-Aldrich, United Kingdom, were prepared for the pH adjustment. Sulfuric acid (H_2_SO_4_), fresh banana peels and distilled water were all used for the experiments, and 1,5-diphenylcarbohydrazide, methanol (HPLC-grade) and a sulfuric acid solution (98%), all purchased from Sigma-Aldrich, United Kingdom, were used to prepare the reagent for the measurement of the concentration of Cr(VI) based on the procedure described in [21].

### 3.2. Preparation of the Adsorbents

Fresh banana peels recovered from Cavendish bananas (*musa acuminata*), sourced from Tesco, underwent a meticulous cleansing process with distilled water before being skillfully sliced into petite fragments. Subsequently, these sliced peels were subjected to a judicious drying procedure in an oven maintained at 65 °C for a duration of 24 h. Following the drying phase, the banana peels were finely crushed into a powder and then securely stored in air-tight seal bottles for future utilization and analysis, constituting what we referred to as dried banana peel powder (DBPP).

#### Preparation of the Acid-Modified Banana Peel Powder (ABP)

The process continued with the creation of the acid-modified banana peel powder (ABP), involving a series of experiments meticulously designed to optimize the yield and effectiveness of the acid-modified peel adsorbent. These experiments explored various reaction temperatures (ranging from 50 to 90 °C) and reaction times (spanning from 5 to 24 h). A 50% sulfuric acid solution was meticulously prepared by diluting concentrated sulfuric acid in a 1 L volumetric flask. Subsequently, 100 mL of this diluted acid was carefully introduced into a 200 mL conical flask, already containing 10 g of pre-weighed DBPP.

The ensuing mixture underwent a reactive transformation under agitation, in accordance with the designated temperature and duration parameters. Following the completion of the reaction, the resulting slurry underwent filtration, and the collected residue was diligently washed with distilled water until achieving a neutral pH. The washed residue was then air-dried in a fume cupboard for a thorough 72 h duration. Subsequently, the yield of the product and its efficacy in the removal of Cr(VI) were comprehensively evaluated (see Supporting Information for full details). Upon analyzing the results, it was determined that a reaction time of 5 h at a temperature of 90 °C proved to be the optimal conditions for the production of the ABP.

### 3.3. Sample Characterization

The functional groups in each sample were identified using Fourier transform infrared (FTIR) spectroscopy within the range of 4000–600 cm^−1^ for before and after adsorption. The surface area and porosity information for each sample were determined by a Brunauer–Emmett–Teller (BET) analysis at 77 K using a Micromeritics Tristar 3020 instrument (Micrometrics, Norcross, GA, USA) with an N_2_ gas flow. A CHNS elemental analysis was used to determine the composition of the carbon (C), hydrogen (H), nitrogen (N) and sulfur (S) in a Perkin Elmer CHNS analyzer PE2400CHNS ( Perkin Elmer, UK). During the analysis of the sample, approximately 2 mg was combusted at a temperature of 975 °C. The textural properties of the samples were determined by scanning electron microscopy (SEM) using an FEI Quanta FEG Environmental SEM (Fisher Scientific, UK) equipped with energy dispersive X-ray analysis. The samples were coated with gold and vacuumed (for 5–10 min) prior to analysis.

### 3.4. Adsorption Experiments

#### 3.4.1. Adsorption of the Cr (VI)

The ABP sample made was used for the adsorption of the Cr (VI). A synthetic stock solution of chromium (VI) was made by dissolving the required mass of K_2_Cr_2_O_7_ in 1 L of deionized water. Then, the Cr (VI) solutions were made, ranging from 20 ppm to 200 ppm. We placed 0.02 g of treated banana peels in 20 mL of each Cr (VI) solution and left them for three days at room temperature. After adsorption, the residual concentrations of the solutions were determined by UV–visible spectrophotometry using the procedure outlined in [21]. The chromium concentrations in the solid phases were calculated using the following equation:

(6) $$q_{e}=\frac{\left(C_{i}-C_{e}\right)\;V}{m},$$

where $C_{i}$ and $C_{e}$ are the liquid-phase concentrations of chromium initially and at equilibrium, respectively, and both were measured in mg/L; V is the volume of the solution (L); and m is the mass of the dry adsorbent used for the experiment in grams. The removal of the Cr (VI) from the solution was calculated using the following equation:

(7) $$\%R=\frac{\left(C_{i}-C_{e}\right)\;}{C_{i}}\;100,$$

where $C_{i}$ and $C_{e}$ are the liquid-phase concentrations of chromium initially and at equilibrium, respectively, and both were measured in mg/L.

To determine the optimum dosage of the ABP adsorbent, different amounts of the ABP were contacted with 20 mL of Cr (VI) solutions with concentrations of 100 ppm over a 24 h period.

#### 3.4.2. Data Analysis—Isotherm Models

(I) Data derived from various isotherm models, such as the Langmuir, Freundlich, Temkin, Redlich–Peterson and Sips isotherm equations, can provide insights into the sorption processes, surface properties, and affinities of sorbents. These models are employed to describe equilibrium data. The suitability of the isotherm models was assessed through the examination of their correlation coefficients [40], which helped in determining their applicability.

The Langmuir isotherm model can be expressed by the following non-linear equation:

(8) $$q_{e}=\frac{q_{max}k_{L}C_{e}}{1+k_{L}C_{e}},$$

where $q_{max}$ (mmol/g) is the adsorbate ion uptake per unit mass of the adsorbent, which is correlated to the adsorption capacity, and $k_{L}(L/mol)$ is the Langmuir constant that is exponentially proportional to the heat of the adsorption and related to the intensity of the adsorption.

Moreover, when using the Freundlich equation, it is assumed that a decrease in sorption energy follows an exponential pattern as the adsorption sites on an adsorbent reach full saturation. This theoretical framework is particularly suitable for explaining the process of adsorption on surfaces that exhibit heterogeneity and involve interactions between the adsorbed molecules. Equation (9) represents the non-linear expression of the Freundlich equation, as follows:

(9) $$q_{e}=k_{F}C_{e}^{\frac{1}{n}}.$$

In Equation (9), $k_{F}$ (L/mmol) is the Freundlich adsorption constant reflecting the relative adsorption capacity of the adsorbent, while 1/n represents a constant signifying the sorbate’s adsorption intensity on the sorbent or the surface’s level of heterogeneity. A lower 1/*n* value indicates a more heterogeneous adsorbent surface.

The Redlich–Peterson isotherm is expressed as follows:

(10) $$q_{e}=\frac{q_{max}B_{pb}C_{e}}{1+B_{pb}^{\beta }C_{e}^{\beta }}.$$

In Equation (10), *B_pb_* is the Redlich–Peterson isotherm constant (L/mmol), $q_{max}$ is the maximum loading achieved and *ß* is an exponent that lies between 0 and 1.

The Sips isotherm model is given by the following:

(11) $$q_{e}=\frac{q_{max}b_{s}C_{e}^{m}}{1+b_{s}C_{e}^{m}},$$

where $b_{s}$ is the Sips constant, which is indicative of the adsorption affinity, m is a dimensionless parameter that qualitatively characterizes the heterogeneity of the adsorbent–adsorbate system and $q_{max}$ is a parameter indicative of the loading capacity of the adsorbent.

The Temkin equation is written as follows:

(12) $$q_{e}=Bln\left(AC_{e}\right).$$

In Equation (12), the constant *B* can be written as *B* =$\;\frac{RT}{b}$, where *b* is related to the heat of the adsorption. The Temkin equilibrium binding constant, A (L/mmol), corresponds to the highest binding energy.

### 3.5. Desorption Study

The ABP sample loaded with Cr (VI) was washed repeatedly with distilled water and dried in an oven at 60 °C for 24 h. Then, it was desorbed in distilled water and 2 mol/L of diluted sulfuric acid for one day and, finally, filtered. The dried, spent adsorbent was contacted with different desorption solutions (deionized water and 2N H_2_SO_4_) and agitated for time periods that were longer than the equilibrium time. We tested for the presence of Cr(VI) in the desorption media using UV-vis. The desorption efficiency was determined by the following equation:

$$Desoprtion\;\left(\%\right)=\frac{amount\;of\;Cr\left(VI\right)ions\;desorbed\;to\;the\;desorption\;medium}{amount\;of\;Cr\left(VI\right)ions\;adsorbed\;to\;the\;ABP}\times 100\%$$

## 4. Conclusions

This study developed an adsorbent from banana peel treated with chemicals to remove chromium ions from water. The kinetics showed that the adsorbent was fast, removing 80% of the chromium in 4 h. Higher temperatures sped up the removal but did not change how much the adsorbent could hold. The desorption tests showed that the adsorption was stable under a neutral pH, preventing chromium from leaking back into the water. The adsorbent had a similar capacity to other materials in the literature. Thus, this adsorbent could be a low-cost alternative for chromium removal.

## Figures and Tables

**Table 1:** BET surface area data of the BP and ABP samples.

	BP	ABP
Surface area (m^2^/g)	0.0363	0.0507

**Table 2:** Elemental analysis results (CHNS) of the BP and APB samples.

Element	BP	ABP
Carbon/%	38.78	38.18
Hydrogen/%	5.71	6.20
Nitrogen/%	1.98	1.18
Sulfur/%	<0.3	4.92
C:N ratio	19.59	32.36

**Table 3:** Summary of the main FTIR peaks for the BP and ABP samples.

Major Functional Groups	BP	ABP
O-H stretch vibration	3400	3424
C=O stretching vibration in a carboxyl group	1730	1730
The asymmetric vibration of C=O in -COO-	1592	1613
The symmetric vibration of C=O in -COO-	1375	1440
C-OH stretching vibration	1023	1029

**Table 4:** Summary of the elemental composition of the ABP sample before and after adsorption.

Element	Relative Composition on Surface (%)	
	Before	After
C	39.6	58.0
O	32.8	39.0
S	15.7	1.5
K	6.1	-
Fe	3.3	0.3
Cr	-	0.6
N	0	-
Si	2.4	-

**Table 5:** Summary of the isotherm model parameters obtained from the non-linear regression fits.

Isotherm Model	Equation	Parameter	Value
Freundlich	$q_{e}=k_{F}C_{e}^{1/n}$	*k_F_* (mmol/g)	5.226
		*n* (-)	2.609
		*R* ^2^	0.956
		*RSME*	0.250
Langmuir	$q_{e}=\frac{q_{max}K_{L}c_{e}}{1+K_{L}c_{e}}$	*q_max_* (mmol/g)	3.867
		*b* (L/mmol)	19.286
		*R* ^2^	0.982
		*RSME*	0.162
Redlich–Peterson	$q_{e}=\frac{q_{max}B_{pb}c_{e}}{1+B_{pb}^{\beta }c_{e}^{\beta }}$	*q_max_* (mmol/g)	2.503
		β (-)	0.850
		*B_pb_* (L/mmol)	42.838
		*R* ^2^	0.984
		*RSME*	0.153
Sips	$q_{e}=\frac{q_{max}b_{s}c_{e^{m}}}{1+b_{s}c_{e}^{m}}$	*q_max_* (mmol/g)	4.510
		*m* (-)	0.782
		*b_s_* (L/mmol)m	12.209
		*R* ^2^	0.985
		*RSME*	0.145
Temkin	$q_{e}=q_{T}ln(K_{T}c_{e})$	$K_{T}$ (L/mmol)	273.673
		$q_{T}$ (mmol/g)	3315.165
		*R* ^2^	0.983
		*RSME*	0.155

*q_e_* is the concentration of Cr(VI) in solid phase at equilibrium; *C_e_* is residual concentration of Cr(VI) *q_max_* is Langmuir adsorption capacity; *K_F_* is the Freundlich partitioning constant; *n* is dimensionless parameter; *β* is dimensionless parameter in Redlich-Peterson equation; *b_s_* is the equilibrium constant; m is dimensionless parameter; *q_T_* is surface capacity for contaminant adsorption per unit binding energy; *K_T_* is Temkin parameter; *RSME* is the root mean square error.

**Table 6:** Comparison of the performances of the current materials with other similar materials in the literature.

Adsorbent Material	Adsorption Capacity [mg/g]	Reference
Dolochar	5.21	[22]
Coffee dusts	39	[7]
Tea dusts	44.9	[7]
CeO_2_@starch particles	48.52	[23]
Bio-waste granules	64–73	[24]
Magnetic bleached tea waste	80	[5]
Chemically modified olive stone	82.63	[24]
Banana peel	90	[16]
Date pits	96.02	[21]
Banana peel	131.56	[17]
Acid-modified banana peel (ABP)	161	Current study

**Table 7:** Summary of the kinetic model parameters obtained at the different temperatures.

Kinetic Model	Parameter	Temperature		
		293 K	303 K	323 K
Pseudo-first-order	*k*_1_ (h^−1^)	0.24 ± 0.03	0.28 ± 0.03	0.52 ± 0.07
	$q_{max}$ (mmol/g)	1.77 ± 0.07	1.82 ± 0.07	1.88 ± 0.06
	*R* ^2^	0.990	0.990	0.987
	*RSME*	0.060	0.061	0.066
Pseudo-second-order	*k*_2_ (g mmol^−1^ h^−1^)	0.13 ± 0.06	0.17 ± 0.08	0.37 ± 0.20
	*q_max_* (mmol/g)	2.08 ± 0.22	2.09 ± 0.20	2.07 ± 0.18
	$h=k_{2}q_{max}^{2}$	0.562	0.743	1.585
	*R* ^2^	0.996	0.959	0.940
	*RSME*	0.118	0.120	0.141

*k*_1_ and *k*_2_ are the rate constants in the pseudo-1st order and pseudo 2nd order equations; *q_max_* is the amount of adsorbate adsorbed at equilibrium; *h* is the initial rate of adsorption in the Pseudo 2nd order equation; *RSME* is the root mean square error.

**Table 8:** Thermodynamic parameters for the adsorption of the Cr^6+^ by the APB at the different temperatures.

∆H° (kJ/mol)	∆S° (J/mol K)	∆G° (kJ/mol)		
		20 °C	30 °C	50 °C
20.6	77.6	−2.21	−2.96	−4.54

## Data Availability

Data are contained within the article and Supplementary Materials.

## Supplementary Materials

The following supporting information can be downloaded at: https://www.mdpi.com/article/10.3390/molecules29050990/s1, Figure S1: Comparison of ABP adsorption performance under different modified conditions After comparison of the control group, the preparation conditions of ABP used in subsequent experiments were 90 degrees Celsius and the reaction time was 5 h. Figure S2: SEM-EDX data of the sample before adsorption. Magnification was 30,000 and acceleration voltage of 20 keV used. Figure S3: SEM-EDX data of the sample after adsorption. Magnification was 30,000 and acceleration voltage of 20 keV used. Images taken in Low vacuum mode to enhance the quality of the images due to slight charging of the sample.
