# Supplementary material for: Kinetics and Thermodynamics Study on Removal of Cr(VI) from Aqueous Solutions Using Acid-Modified Banana Peel (ABP) Adsorbents
Source: Molecules. 2024 Feb 24;29(5):990. doi: 10.3390/molecules29050990 (PMC10935434; doi:10.3390/molecules29050990)
Supplement: Supplementary file 1 [file molecules-29-00990-s001.zip › molecules-2773202-supplementary.pdf]

Kinetics and Thermodynamics study on removal of Cr(VI) from aqueous solutions using acid-modified banana peel (ABP) adsorbents

Zhouyang Haung, Robyn Campbell and Chirangano Mangwandi*
School of Chemistry and Chemical Engineering, Queen's University Belfast, David Kier Building
Stranmillis Road, Belfast BT95AG, Northern Ireland, United Kingdom.
E-mail: c.mangwandi@qub.ac.uk

Supporting Information

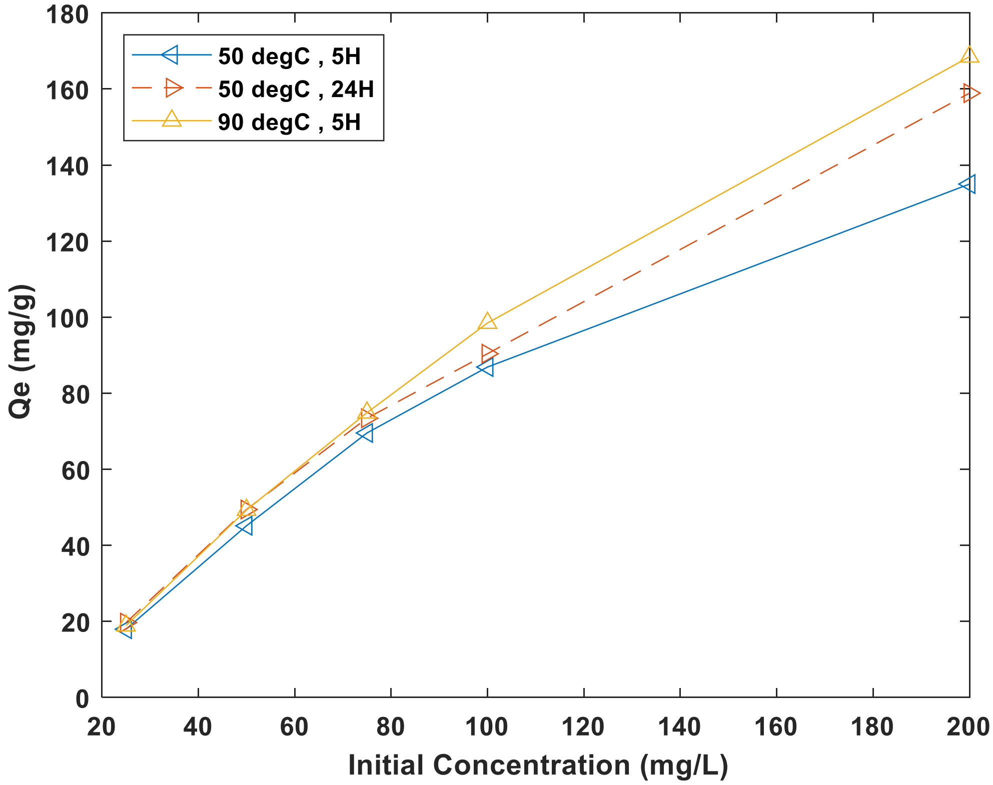

Figure S1 Comparison of ABP adsorption performance under different modified conditions
After comparison of the control group, the preparation conditions of ABP used in subsequent experiments were 90 degrees Celsius and the reaction time was 5 hours.

SEM -EDX Data 30k Magnification before Adsorption:

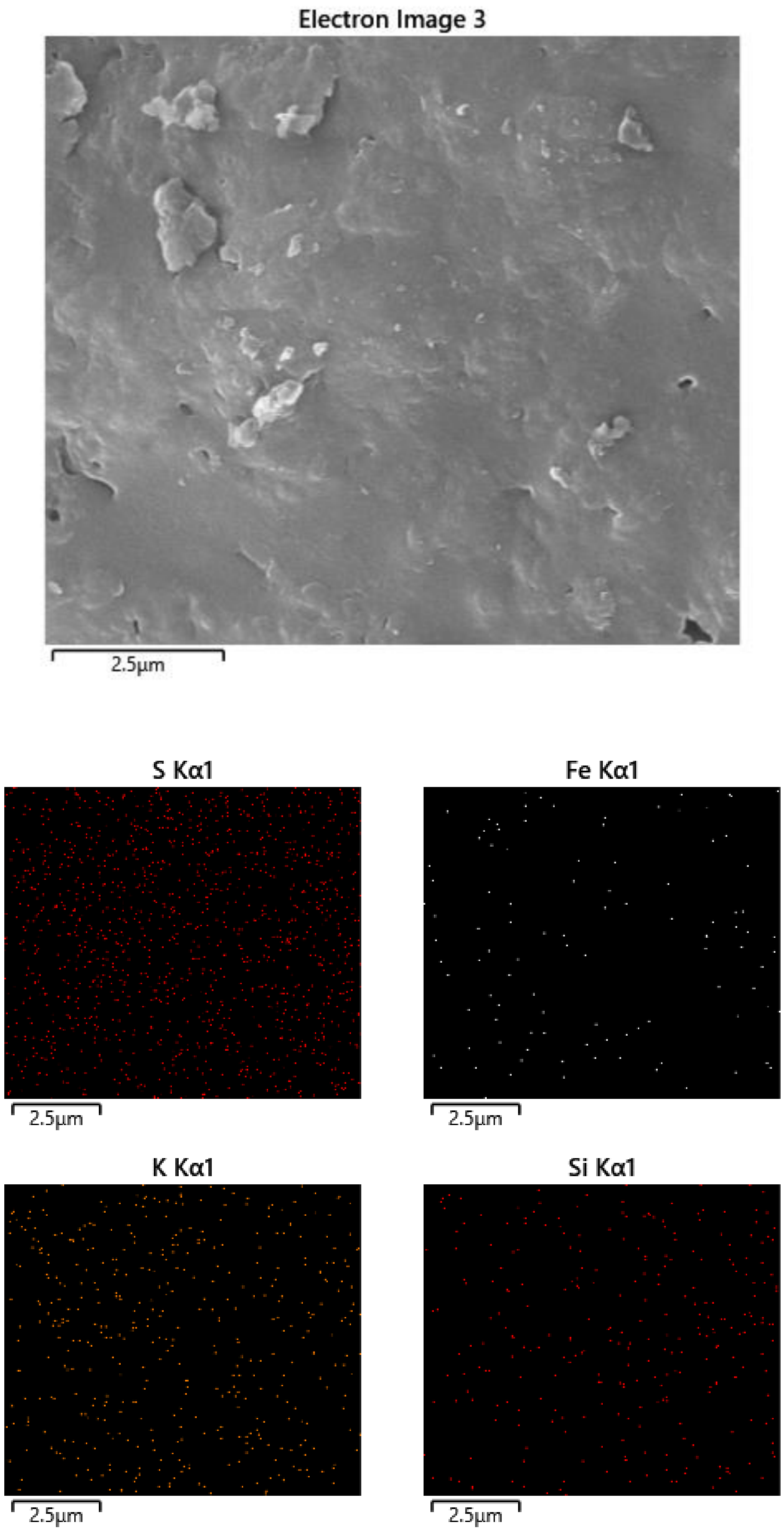
Electron Image 3
2.5µm
S Kα1
2.5µm
Fe Kα1
2.5µm
K Kα1
2.5µm
Si Kα1
2.5µm

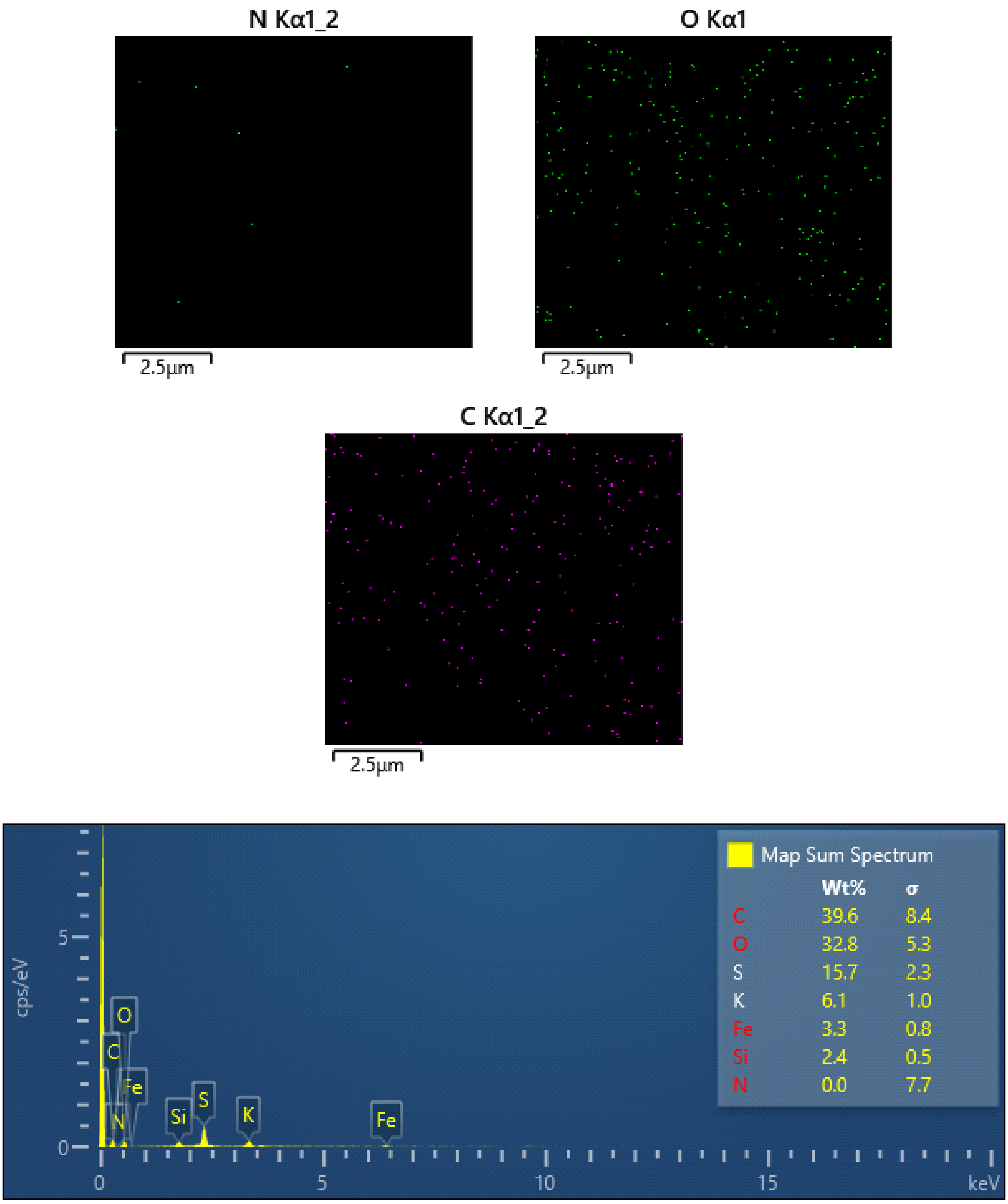

Figure S2 SEM-EDX data of the sample before adsorption. Magnification was 30000 and acceleration voltage of 20 keV used.

Electron Image 5

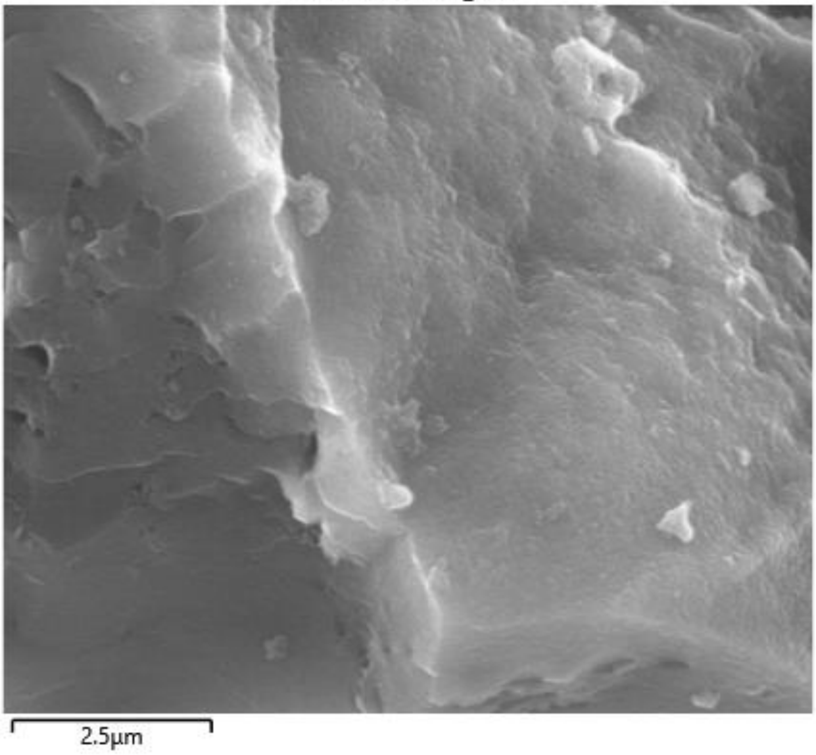

S Kα1

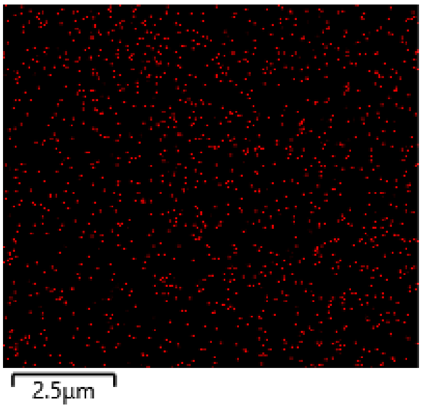

O Kα1

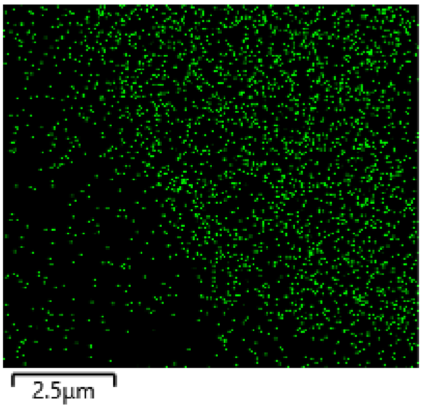

C Kα1_2

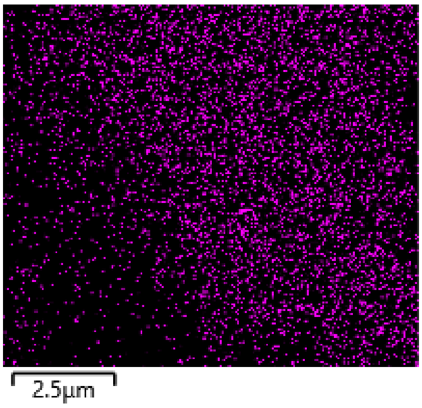

Fe Kα1

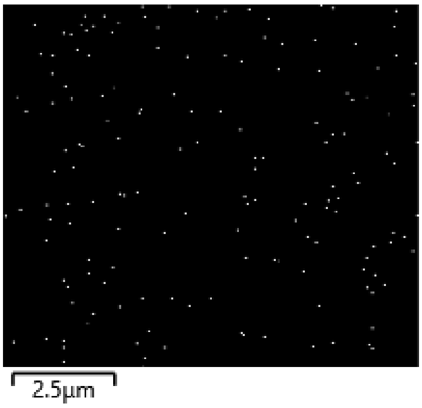

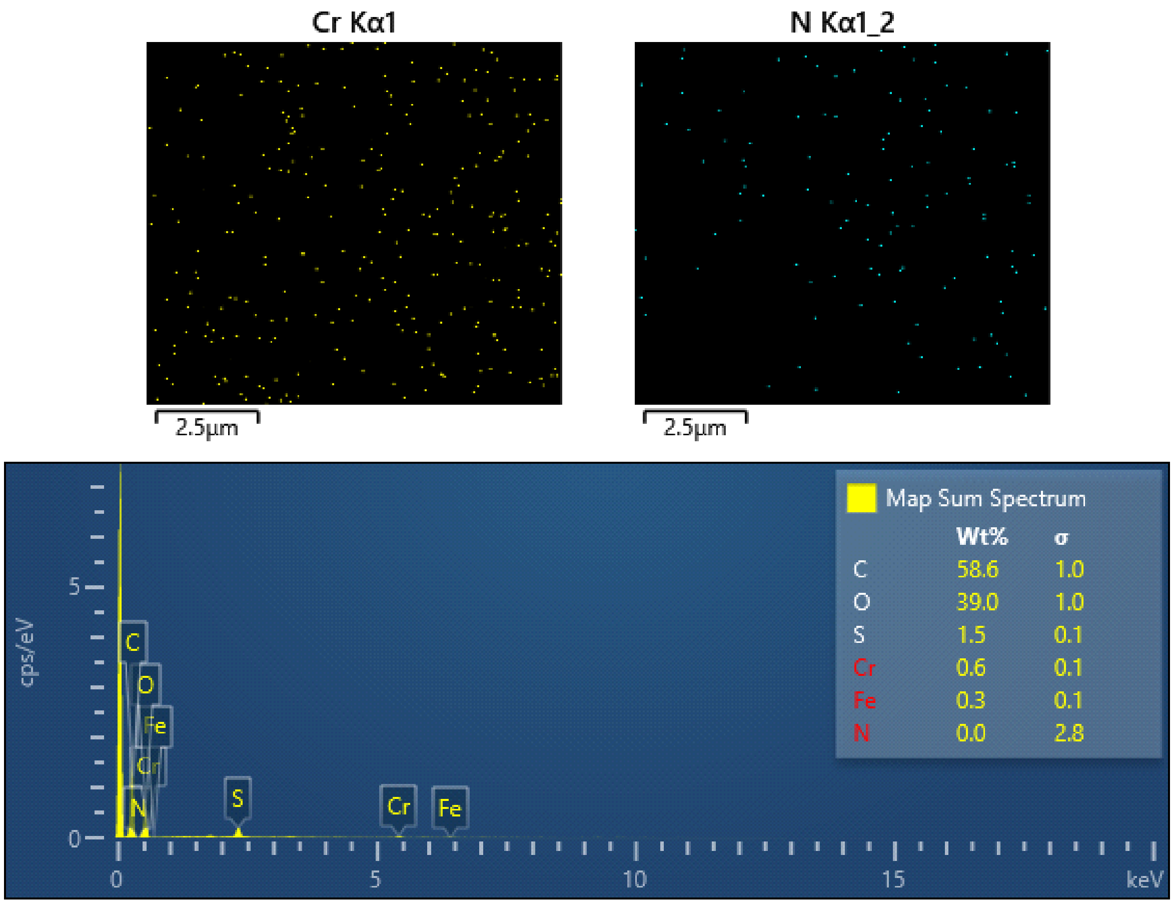

Figure S3: SEM-EDX data of the sample after adsorption. Magnification was 30000 and acceleration voltage of 20 keV used. Images taken in Low vacuum mode to enhance the quality of the images due to slight charging of the sample.
